# Intimate Partner Violence and its Escalation Into Femicide. Frailty thy Name Is “Violence Against Women”

**DOI:** 10.3389/fpsyg.2018.01777

**Published:** 2018-09-26

**Authors:** Georgia Zara, Sarah Gino

**Affiliations:** ^1^Department of Psychology, University of Turin, Turin, Italy; ^2^Laboratory of Criminalistic Sciences “Carlo Torre, ” Department of Public Health and Pediatrics, University of Turin, Turin, Italy

**Keywords:** violence, intimate partner violence, intimate relationships, femicide, overkilling, risk

## Abstract

Violence against women is a disabler of dignity, liberty, and rights of the person, with murder being its extreme form for silencing the individual. Despite psycho-criminological research providing evidence that violence can happen across cultures, sexes, and societies, other findings show that some forms of violence i.e. Intimate Partner Violence (IPV), which involves more frequently women as victims, is not rare in contemporary society. The aim of this study is to analyze the violence against women, and how it escalates up to the point in which it aggravates into femicide. In order to carry out this study, data from both the Turin Archive of the Institute of Legal Medicine (1970–1997), and the Archive of the Central Morgue (1998–2016) were collected. The interest was to focus on those women who were killed in Turin, between 1970 and 2016, by a male with whom they were involved in a more or less intimate relationship (e.g., matrimonial, sexual, friendship, professional, etc.). Collateral information was also gathered from forensic files that reported sufficient details about the criminal events. The sample was composed of 275 women killed by violence in Turin, Italy, by 260 males. This research was based on two questions: Is murder the worst possible scenario of a long-lasting abusive relationship? Are we witnessing a shift in how violence now happens, becoming perhaps less striking than murder, but not less painful from the victim's point of view? These findings show that escalation into femicide featured more likely within an intimate and affective relationship between victim and perpetrator; they also show that when the perpetrator knew the victim, it was more likely that an overkilling took place. When victims sustained multiple injuries that went beyond those necessary to cause their death, one is in front of an overkilling. These results also suggest that motives behind intimate partner femicide could account for a differential degree of violence, so that the longer and closer the relationship was between victim and perpetrator, the higher the risk of IPV escalating into femicide, and of femicide being executed with extreme and severe force.

## Introduction

Violence is a disabler of dignity, liberty, and rights of the person, with murder being its extreme form for silencing the individual. The focus here is on violence against women that leads to femicide.

Violence against women (VAW) can take many forms and shapes; it can be acted out through behavior or it can be psychological, and therefore difficult to be seen or measured. It can be enduring and last for long periods, or it can be brief, but nonetheless intense. VAW is one of the highest concerns global-wise, and much attention, resources and sensitivity are required to put in motion coordinated strategies to intervene to stop its escalation and its worsening.

Though studies are suggesting that violence is not exclusive to men, but that women could also be perpetrators (Sommers, 1994; Ristock, 2002; Belknap et al., 2012), data from the World Health Organization (WHO) are clear in suggesting that women are more likely than men to be the target of violence (Stewart, 2002; ISTAT, 2006), of sexual abuse (Zara, 2018), of psychological abuse (Pico-Alfonso, 2005), of domestic violence (Johnson and Ferraro, 2000; Lloyd et al., 2017), of intimate partner violence (Theobald et al., 2016). This could be also a result of women becoming more open about the problems than men, hence they are more likely to report the abuse and violence than men are (Dutton, 2010), and this may be more so the younger the people involved (Capaldi et al., 2012; Kim et al., 2016).

The most common form of violence suffered by women is intimate partner violence (IPV) (World Health Organization, 2013). IPV is a global concern (Butchart et al., 2010). IPV occurs in different settings, across socio-economic classes, cultures, ages, religious groups (Archer, 2006). In its extreme forms, IPV causes death (Campbell et al., 2007).

Available definitions of IPV are diverse, often either too inclusive or too specific not allowing for a comprehensive perspective. Not all forms of abuse that women suffered from occur within domestic life. Nor can IPV always be explained by gender violence.

Looking at some of the major studies carried out in this field (e.g., Dutton and Goodman, 2005; Stark, 2007; Campbell et al., 2008; Johnson, 2008; Hart and Klein, 2013), Hart and Klein (2013) suggest that IPV endorses physical, sexual, psychological, economic abuse and stalking that are the five multi-faceted methods of violence and abuse that perpetrators utilize to achieve, maintain and regain control of their intimate partners or potential ones. Coercion or terroristic threats, coupled with any of the five methods of abuse above mentioned, constitute IPV (Smith Slep et al., 2016).

Intimate relationships are defined as relationships that involve an intense emotional and/or physical investment (Miller, 2015). Intimacy is a primary human need (Strong et al., 2011), and the need to belong is a fundamental human motivation that fosters desire for interpersonal attachments (Baumeister and Leary, 1995). This is why, Baumeister and Leary (1995) advocate that “much of what human beings do is done in the service of belongingness” (p. 498). Human companionship strongly affects the quality of life (Robles et al., 2014), as well as influencing rates of illnesses, such as cancer, and mental well-being (Whisman et al., 2010). This is no surprise since studies show that people who experience a fulfilling relationship or marriage are generally healthier and have lower mortality rates than divorced, separated, and never-married individuals (Strong et al., 2011).

The intense emotional investment in a relationship could also be imaginary when a person thinks that the other partner is reciprocating and is interested in developing closeness and physical proximity too. According to the *Encyclopedia of Human Relationships* (Reis and Sprecher, 2009) relationships are fundamental to nearly all domains of human activity along the life-course. In the broad concept of human relationships are included all types of human associations, friends, lovers, spouses, room-mates, work colleagues, team-mates, parents and children, relatives, neighbors, business partners, and so forth. Although each of these connections is unique in some respect, they share a common core of principles and processes. When people are involved in healthy, satisfying relationships, they live, work, and learn more proficiently. When relationships are distressing or frustrating, or too asymmetric in the types and quality of interaction, people are less satisfied, less healthy, and less constructive. Dysfunctional relationships are often at the basis of abuse, violence, homicide and femicide (Shackelford et al., 2005).

Violence does not always, or immediately, lead women to death, but the consequences of these acts are equally debilitating; the physical, psychological and social effects of violence vary. It has been established that VAW incurs high costs^1^, including both those related to preventing or dealing with this type of violence (such as police, risk assessment practice, law enforcement)^2^, and those costs incurred by its consequences (such as health) (Varcoe et al., 2011).

### The scientific focus of IPV and femicide

Scientific, clinical and social concerns directed at violence against women, and at how relationships between men and women escalate into femicide have generated significant world-wide initiatives to attempt to intervene efficiently enough to prevent it from its buds or to stop its persistence, escalation and aggravation.

The term femicide was first used in 1801 by Corry in his—*A Satirical View of London at the Commencement of the Nineteenth Century* (p. 49)—intended to signify the killing of a woman. His description of this species of delinquency (as he put it) was also influenced by the cultural and moral tone of the period, though not diametrically opposed to many of the motives behind IPV nowadays. This is confirmed by the fact that it was also recognized that women could perform violence and murder (Corry, 1801/1809, p. 168).

Nearly two centuries after, femicide was used to symbolize a gender-based murder (Russell and Harmes, 2001; Russell, 2012). The term includes not only female murders committed by partners or former partners, but also of girls murdered by their fathers or relatives because they rebel against an obsessive control of their lives, identities or sexual choices, or because they refuse a marriage imposed to them (Russell and Harmes, 2001). The term also includes any murder of a woman by a man, independently of the type and intensity of the relationship, because of the exercise of power or dominance, for reason of hate, disdain, passion or sense of ownership over the same woman.

The social and ideological mainstream stance that VAW and femicide research has taken (Bandelli, 2017) fosters the assumption that if one investigates closely the types of perpetrators and victims, and their relationships, one betrays the social and cultural nature of it. Conversely, scientific research should fight against any form of reductionism, if the endeavors are to grasp complexity, and to avoid moral panic. The former requires bringing VAW into focus by offering an integrated perspective in which individual, relational and social dimensions are not artificially separated. The latter looks critically at any delimitation in this field of knowledge that “lay down the rules by which the problem can be talked about” (Critcher, 2003, p. 168).

The separation of the individual from the social setting is not recent, and many eras in history have witnessed this hiatus. Donati (2011) argues that “today matters are different because they are no longer simply instances of dehumanization, but of an irruption of the inhuman into the social, one that progressively displaces what is still human” (p. 21). By doing so, the risk is “that the ‘social’ is no longer seen or heard […] as something immediately human” (Donati, 2011, p. 21), and it becomes not anymore related to the individual reality, and to how the person contributes to promote or endures it.

Few studies have examined these aspects and played a significant part in expanding our understanding, and enlightening the processes that can trigger interpersonal and intimate violence into women targeted violence, and from this into femicide. It is interesting to see that some of these studies embrace either a forensic and legal medicine approach or a psycho-criminological and risk assessment approach.

For the former approach, it is worth mentioning three recent studies carried out in Italy. Bonanni et al. (2014) investigated the Italian scenario of femicide by analyzing four cases chosen to profile a specific sub-group of femicide, and comparing it with the international one. Data regarding the type of relationships victims and perpetrators had before the killing took place, what still bound them to the point of the extreme act of killing, and whether the victim's decision to break off the relationship represented the trigger for the rage into femicide, were examined. It emerged that the prior relationship between perpetrator and victim was relevant in the cases investigated, and also influenced the modus operandi by which women were killed.

Moreschi et al. (2016) explored the cases of 34 femicides that occurred in Italy over a 21-year period (1993–2013). Besides the analyses of typical epidemiological aspects of femicide, the focus was on the circumstances and risk factors surroundings the crime, and through the examination of aspects such as perpetrator's motive or specific risk factors (e.g., legal possession of firearms, previous violence and threats, time occurred after the ending of the relationship), their study aimed at profiling some possible preventive strategies.

Another work on femicide in north-west Italy carried out by Trecastagne et al. (2016) integrated a forensic pathology approach with a more social perspective in order to establish to what extent the law and the cultural changes, which took place in Italy between 1970 and 2012, had an impact on the rate of these crimes.

For the psycho-criminological and risk assessment approach, some specific studies look at the risks involved in VAW and in IPV. Owing to the seriousness and prevalence of the problem, Kropp and Cook (2014) believe that it is essential to provide the criminal justice system, the court, the health care, and shelter and protection settings with scientific evidence on how to conduct risk and threat assessment, and which instruments to use, with whom, when, and in which contexts. Most IPV risk assessment tools^3^ involve an integrated assessment of criminogenic risks of offenders, along with an evaluation of the victimogenic risks of the victims (Campbell et al., 2001; Baldry and Winkel, 2008). When assessing the risk of IPV, it seems in fact partial not to explore the criminogenic dimension along with the victimogenic one.

Other studies have attempted to explore intimate partner violence by looking longitudinally at the risk processes implicated. Lussier et al. (2009) analyzed data from the prospective longitudinal *Cambridge Study in Delinquent Development* (CSDD) to examine to what extent IPV in mid-adulthood could be predicted by early childhood risk. Neuropsychological factors (e.g., verbal reasoning, verbal intelligence, etc.) and a criminogenic family environment (e.g., parental criminal record, low income, inadequate parenting, parental conflict, etc.) were measured between ages 8 and 10, while antisocial behavior was measured from age 8 to age 18 (e.g., overt behavior such as aggression and violence; covert behavior such as being deceitful and dishonest; reckless behavior; authority-conflict behavior). IPV was measured at age 48 using a self-report instrument completed by the participants' female partners. Findings suggest that perpetration and victimization rates were relatively high; violence was mostly mutual, and men were more likely to be victims than perpetrators. A criminogenic environment increased the risk of IPV by fostering the development of antisocial behavior and neuropsychological deficits, suggesting that IPV is never a private matter, but that the nature and quality of the relationship, along with individual and familial factors, contributed to IPV. Other studies using CSDD data advocated that processes of discontinuity and continuity between childhood and adolescent risk factors seem to increase the likelihood of future involvement in IPV by male partners (Theobald and Farrington, 2012), and between generations, though with some differences in risk factors for males and females (Theobald et al., 2016). Hence, the acknowledgment that IPV is not just situational, contextual and cultural is scientifically recognized.

Moving in this direction, the aim of this paper is to examine critically the individual and the social, the psychological and the relational, the cultural and the human aspects of what makes people violent, what encourages men to abuse women, and what fosters male partners to kill their female partners. The assumption is that a one-factor explanation i.e., patriarchal culture (Walker, 1989) and the reinforcement of its values (Ehrensaft et al., 2004; Dutton and Nicholls, 2005), cannot provide for comprehensive and exhaustive explanations (Noller, 2007) of what triggers IPV. IPV does not occur in a social, relational, and psychological vacuum: it is likely that the type and quality of the relationship might play a significant role in setting up opportunities to exercise aggression, and direct violence. It follows that an integrated approach might foster a broader and deeper understanding of what makes people violent, and what makes men abusive and aggressive toward women.

### The present study

This paper addresses intimate personal violence, and differentiates between risk factors that are at the basis of what triggers violence against women, and what sustains it in time, by an integrated and interdisciplinary perspective. The type, intensity, and length of relationships between female victims and male perpetrators will also be looked at, so as to be able to explore the extent to which IPV could contribute to femicide.

The focus of this study is on IPV, and whether its forms have changed over the years, and if yes, how. The temporal period of investigation is 46 years (1970–2016), which is a sufficiently long period to allow for exploring possible changes in the type of victims targeted; the type of relationships and the affective intensity between victim-offender; the dynamics involved in the perpetration of violence up to the escalation into femicide. Understanding VAW during this temporal period could offer insights into those early risk factors that influence and alter the quality of interpersonal and intimate relationships, and that may be informative to endorse preventive interventions before it would be too late.

## Materials and methods

### Hypotheses

IPV is likely to occur within a relationship between victim and perpetrator, and not independently of it.

It is assumed that the heterogeneity that featured in how violence is perpetrated depends at least on two aspects: the type of relationships between female victims and male perpetrators, and motives for being violent. These aspects affects how violence is performed, and the extent to which it escalates. While it is accepted that violence is a matter of individual (criminal) responsibility, it is also important to analyze the context in which VAW occurs, so as to be able to identify those risk factors, and relational and social conditions that make it possible for perpetrators to abuse and kill their victims.

It is assumed that not all VAW are gendered targeted. It is assumed that IPV is likely to be addressed toward specific victims with whom the perpetrator has had, or has, or wished to have (or have had) a relationship. The “relationship component” is likely to confer to the violence a particular overtone in the dynamic of killing, in the weapons used, and in the setting in which it occurs.

It is assumed that the closer and more intense the relationship between perpetrator and victim is, the more brutal the violence is, and the more likely that it will escalate into overkilling.

### Procedures

In order to meet all the ethics standards, the researchers in this study followed all possible procedures to ensure confidentiality, fair treatment of data and information, and to guarantee, at each stage of the research, that the material was treated with respect and discretion. The research protocol was organized according to *The Italian Data Protection Authority* (Garante per la protezione dei dati personali), nr. 9/2016, artt. 1 and 2 (application and scientific research purposes), and art. 4 (cases of impossibility to inform the participants, e.g., deceased people), and in line with the Italian and the EU code of human research ethics and conduct in psychology, forensic pathology and legal medicine.

While valid consent is a paramount requirement in scientific research with human participants, there in fact can be an exception to exempt researchers from obtaining consent. This is the case in which participants are deceased. This research lies in this specific situation because the sample comprises femicide victims. The data were archived both at the Institute of Legal Medicine, which signed a letter of intent with the Department of Psychology (University of Turin) to support this research, and at the Archive of the Central Morgue of Turin whose Director authorized data collection.

The research was assessed and approved by the Bioethics Committee of the University of Turin (protocol nr. 191414/2018).

All data were anonymized and made unidentifiable, and were also numerically coded for statistical purposes. The software package *IBM SPSS Statistics* version 25 was used.

Through a retrospective review of database of records from the Institute of Legal Medicine, and the Archive of the Central Morgue in Turin, this study identified all cases of women killed in Turin city and its outskirts from 1970 to 2016.

The sample comprises all female victims by male perpetrators. Features and characteristics of female victims and male perpetrators were taken into consideration. Specifically the types of relationships between victims and perpetrators, whether the relationship was abusive, the dynamics of the killing, and the motives that fostered the escalation into femicide were explored. Injury data were extracted by study investigators directly from the medical examiner records; when available the research team reviewed final autopsy reports and juridical records. Most data gathered contain information about age, race, profession, type and length of relationship between victim and perpetrator, social status, injuries, place of killing, reaction of the perpetrator after the murder, and a final determination of manner of death (natural, injury, murder, suicide), and cause of death. The cases of natural or suicidal deaths of women were excluded from the analysis.

To define injuries as excessive killing or overkilling, conservative scientific criteria were followed, so that if the victims sustained multiple injuries that went beyond those necessary to cause their death, this was accounted as overkilling. Jordan et al. (2010) suggest that overkilling involves multiple injuries resulting in one or more causes of death (i.e., multiple gunshots wounds) or multiple wounds distributed over two or more regions of the body (Salfati, 2003). The body regions were divided into three macro-regions: a. head, neck, face; b. torso and arms; c. legs. The categorization of data into motives of crime i.e., risk factors for intimate partner (IP) femicide, and then assessing whether the femicide comprised overkilling, were completed by two independent raters. When a discrepancy emerged, they discussed the case with the research group, and re-assessed it, until a better level of agreement was reached. The Cohen's Kappa statistic (Cohen, 1960) provides a measure of agreement between raters that takes into account chance levels of agreement, and it is appropriate for this type of data. Kappa for the category ‘motives of crime’ was = 0.782, while for the category ‘overkilling’ Kappa was = 0.778, suggesting, according to Viera and Garrett (2005), a substantial inter-rater agreement coefficient for both variables.

During data collection, every effort was made to record additional information related to the life of victims and of perpetrators so as to be able to reconstruct the events that led to the mortal accident as accurately as possible. Whenever possible data related to previous IPV or domestic violence incidents were recorded. Only a portion of data collected through file reviews are presented here.

### Sample

The final sample involved in this study was composed of 275 women killed by violence in Turin, Italy. The violence was perpetrated by 260 males, indicating that in 95 % of cases the perpetrators (*n* = 247) killed only one victim, while in 11 cases they killed two victims, and in two cases three victims.

For some specific variables (e.g., nationality of the perpetrators and victims, criminal records, types of relationship, crime scenes, etc.) some data were missing; hence, some of the percentages do not sum up to 100%.

The victims were mostly Italian (*n* = 247; 89.8%), while the rest of the victim sample was comprised of foreigners. In 45.4% of cases they were unemployed (*n* = 104); in 28.8% of cases (*n* = 66), they were involved in a non-qualified job (e.g., cleaning job), and in 25.8% of cases (*n* = 59) they held a qualified profession (e.g., nursing, etc.).

While it was not simple to gather complete data on the 260 perpetrators, every possible effort was made to collect sufficient information to profile them. The vast majority of perpetrators were Italian (*n* = 177; 91.2%), while in 17 cases they were foreigners (8.8%). In 29.8% of cases, they were unemployed, while in 54.2% of cases (*n* = 97) they had an occupation. When employed, 46.9% (*n* = 84) of them were involved in a qualified profession (e.g., civil service, teaching, etc.), and 4.7% of them (*n* = 13) had an unqualified profession (e.g., trading, factory work, etc.).

When data were available, their criminal careers were taken into account by looking at whether they had been involved in any previous crime apart from the index offense of femicide they were convicted for. In 77.7% of cases (*n* = 202) it was the first time they were officially involved in an offense, with no previous convictions reported; the rest of the sample was made up of 22.3% (*n* = 58) of individuals who had previous convictions.

### Analytical strategy

Descriptive analyses with Chi-square and Odds Ratio (OR) were carried out to explore characteristics of the sample involved. The OR was calculated to identify which factors significantly explained motives for killing and which others predicted the dynamic for killing up to extreme killing, i.e., overkilling. The OR provides information about the existence, direction, and strength of an association between target and comparison groups regarding the likelihood of an event occurring (Farrington and Loeber, 2000). Where ORs are higher than 1, people with that particular attribute have relatively higher odds of offending than those who do not have this attribute.

## Results

Table 1 synthesizes the historical distribution of women killed in the Turin area every 5 years from 1970 up to 2016. As shown, the numbers of femicides decreased by years, showing a higher concentration of killing incidents up to 1996, and then a decrease. Dividing the crime period at the quartile cut off year (1996), into two macro-temporal categories, these data suggest that 73.5% (*n* = 202) of the female murders occurred from 1970 to 1996 (*dated* femicides), while 24.6% (*n* = 68) took place between 1997 and 2016 (*contemporary* femicides).

**Table 1:** Trend of women victims of murder in Turin by years.

**Historical period**	***F***	**%**
1970–1975	37	13.7
1976–1980	49	18.1
1981–1985	44	16.3
1986–1990	40	14.8
1991–1995	24	8.9
1996–2000	31	11.5
2001–2005	18	6.7
2006–2010	11	4.1
2011–2016	16	5.9
Total	270	100.0

*For five cases it was not possible to establish the year of crime*.

Contrary to the common opinion, which fosters the idea that women are more in danger in isolated places and at night, in 73.9% of cases (*n* = 198) the killing usually occurred in the house of the victims or of the perpetrators, and in 26.1% of cases (*n* = 70) the victims were killed either in an isolated place (e.g., country side or outskirt of the city) or in the car. Most of the deadly incidents (61.5%; *n* = 155) occurred during the day, between 06:00 a.m. and 5:59 p.m., while the rest of the victims (38.5%; *n* = 97) were killed at night, between 6:00 p.m. and 05:59 a.m.

### Vicims and perpertrators: who were they?

In this study, the victims had an average age of 46.15 (*SD* = 20.96), while the perpetrators had an average age of 42.96 (*SD* = 16.60). This difference was near to statistical significance indicating that the victims were slightly older than their offenders, *t*_(448,366)_ = −1.821, *p* = 0.069, showing a small effect size, *r* = 0.17.

In 88.3% of cases (*n* = 228), the victims knew the perpetrators, while in only 11.6% of cases (*n* = 30) the perpetrators were strangers. Looking closely at those cases in which the victims knew their perpetrators, in 60.5% of cases (*n* = 156) they were involved in an intimate relationship; in 27.9% of cases (*n* = 72) they were acquaintances (e.g., neighbors, customers, etc.).

When victims and perpetrators knew each other, the average length of the relationship was 13 years (*SD* = 13.36; Min. = 0.01 month - Max = 62 years). Looking closely at these cases in which they had a relationship, in 43.1% of cases (*n* = 88) they never lived together, while in another 43.1% of cases (*n* = 88) they were living together and shared a house when the killing occurred. In another 13.7% of cases (*n* = 28) the killing occurred after their cohabitation was interrupted. In all cases, there was some evidence that an undergoing dysfunctional relationship mortgaged their life.

### The dynamic of crime

In 75.3% of cases (*n* = 207) the perpetrator responsible for the killing was identified, found guilty, and convicted, while 24.7% of cases (*n* = 68) comprised unsolved and cold cases.

Information about the dynamic of crime and the reaction of perpetrators after the killing was also gathered. Data suggest that in 46.5% of the cases (*n* = 107) the perpetrators reacted by disposing of the victim's body, escaping, or denying the event or having any responsibility in it. In 53.5% of cases, perpetrators admitted the crime. In 13.8% of cases (*n* = 36) the murder was followed by suicide.

The types of lethal weapons used to kill the victims comprised sharp weapons and firearms in 57.5% (*n* = 150), while 42.5% of cases (*n* = 111) involved the use of improper weapons such as objects and bare hands. In those 59 cases in which the information was known, the firearms were possessed illegally or were not clearly registered to the authorities in 54.2% of cases (*n* = 32), though in most of these cases (*n* = 37; 62.7%) the perpetrators seemed to have handled the firearms with easiness, measured by the ratio between tagging the victim target and spotting successfully the target even at distance, and when in movement.

### Dying by which death?

The most common causes of death were gun injuries, stab wounds, and strangulation. The body regions mostly involved in the injuries were in 53.6% of cases (*n* = 140) the head, neck and face of the victims, in the 23.4% (*n* = 61) the upper-body of the victims (e.g., torso, chest, and arms), and in 23.0% of cases (*n* = 60) the injuries were spread over the entire body. In 56.3% of cases (*n* = 103) the victims did not manifest any defense reaction. In 40.7% of cases (*n* = 111) the victims were overkilled, implying that the attack caused excessive trauma beyond that necessary to cause death.

Understanding the motives behind the IPV that led to violent death was also explored. As emerged in previous studies (Campbell et al., 2007), motives are heterogeneous, and difficult to be identified precisely, especially because in most cases concurrent causes are at the basis of the crime. The motives behind the killing of the women involved in this study can be divided into six main categories. In 31.1% of cases (*n* = 80) the motive was crime of passion mostly related to an intense relationship that went terribly wrong; in 18.7% of cases (*n* = 48) the motive was related to family problems that involved ruminative thinking over difficulties such as insurmontable debts, a piece of news over the diagnosis of an incurable disease or losing the job. These events seemed to have preoccupied intensively and obsessively the perpetrator to the point of desperation: in these cases evidence of rows between the victim and the perpetrator sustains the assumption that the killing was not impulsive, but evolved into a reaction that from bad went worst. In 15.2% of cases (*n* = 39) the victim was killed as a consequence of another crime, and the motive was purely antisocial; in 14.0% of cases (*n* = 36) the motive was a predatory crime that involved also some sexual motivated killing. In 13.6% of cases (*n* = 35) the perpetrator acted under impulsivity and loss of control after a row or a refusal, and in only 7.4% of cases (*n* = 19) the perpetrator acted because of a mental disorder. In 18 cases it was not possible to identify the motives of crime.

It was significant to explore whether the types of relationship and the emotional intensity involved between victims and perpetrators (stranger vs. intimacy vs. affective vs. family closeness) were risk factors for an extreme act of killing that led to overkilling, and whether the overkilling could be differently explained by the variety of motives involved.

An analysis carried out with Chi-square and OR explored the likelihood of overkilling by types of relationships and motives of crime.

In 45.1% of cases (*n* = 82), when the victims knew their perpetrator the likelihood of being overkilled, with death being preceded by afflicting and brutalizing acts, was higher in comparison with the likelihood of being overkilled by a stranger (*p* = 0.002). The OR shows that the risk of overkilling almost quintuplicated when the perpetrator was known to the victim, rather than when he was a stranger. Table 2 shows the significant results.

**Table 2:** Overkilling by relationship types and affective intensity.

**Comparing motives**	**Overkilling by relationship types and affective intensity**				
	***F***	**%**	**χ^2^**	***p***	**Odds Ratio (95% CI)**
Unknown victims (0) (*n* = 30)	4	13.3	χ^2^ = 9.474 (*df* = 1)	*p* < 0.002	5.330 (95% CI = 1.788–15.891)
Known victims (1) (*n* = 182)	82	45.1			
Unknown victims (0) (*n* = 30)	4	13.3	χ^2^ = 8.138 (*df* = 1)	*p* < 0.004	4.893 (95% CI = 1.630–14.691)
Intimate/affective victims (1) (*n* = 156)	67	42.9			
Unknown victims (0) (*n* = 30)	4	13.3	χ^2^ = 10.455 (*df* = 1)	*p* < 0.001	6.500 (95% CI = 2.059–20.520)
Non affective victims (1) (*n* = 72)	36	50.0			

*Comparing coding = 0; 1*.

The risk of being overkilled continued to be significantly different depending on the types of relationship involved, and the more intense the emotional closeness between victim and perpetrator.

When the victim was killed by a partner or an ex-partner or a family member, with whom there was respectively an intimate or an affective bond, the risk for being overkilled was almost five times higher than when killed by a stranger. The risk of being overkilled was also significantly higher when there was a sort of relationship, albeit superficial, between victim and perpetrator, as in cases of neighbors, or colleagues or acquaintances, than in those cases in which the victim was killed by a complete stranger. No difference in the level of risk was found when comparing the degree of closeness in the relationship (intimate and affective *versus* acquaintance), indicating that in such cases the women were indifferently killed with a similar violent intensity.

Differences emerged when motives for killing were explored. When comparing crime of passion vs. family problems the likelihood for the woman of being overkilled was significantly higher. Similar risk emerged also when crime of passion was compared with antisociality (e.g., being overkilled as a consequence of another crime being committed) (see Table 3): when the perpetrator was emotionally related to the victim and acted out because of passion, it was more likely that he overkilled the victim. No significant differences emerged when the woman was killed because of predatory violence or because of impulsivity or because of a mental problem of the perpetrator.

**Table 3:** Overkilling by motives for deadly violence.

**Comparing motives**	**Overkilling by motives**				
	***F***	**%**	**χ^2^**	***p***	**Odds Ratio (95% CI)**
Crime of passion (0) (*n* = 80)	43	53.8	χ^2^ = 5.255 (*df* = 1)	*p* < 0.022	0.391 (95% CI = 0.184–0.830)
Family problems (1) (*n* = 48)	15	31.3			
Crime of passion (0) (*n* = 80)	43	53.8	χ^2^ = 5.425 (*df* = 1)	*p* < 0.020	0.351 (95% CI = 0.153–0.802)
Antisociality (1) (*n* = 38)	11	28.9			
Crime of passion (0) compared with: Mental disorder (1); Impulsivity (1); Predatory killing (1) showed no significant results.					

*Comparing coding = 0; 1*.

In order to explore whether, in the course of the 46 years considered, the motives behind violence that led to death changed, crimes committed from 1970 up to 1996 (*n* = 190) were compared with those committed from 1997 up to 2016 (*n* = 62). Findings show that while overkilling was not significantly different depending on the period in which the woman was killed, some differences were found when motives were compared by the two macro-categories considered (multi-problematic relationships vs. antisociality). It was more likely that contemporary femicides (those occurred from 1997 onward) were motivated by multi-problematic relationships rather than general antisociality (OR = 0.576; 95% CI = 0.315–0.1.054). While this result is only near to statistical significance (*p* = 0.09), it suggests that interpersonal, intimate or family problems were likely to drive a person to kill especially if the perpetrator was emotionally closer to the victim, and some emotional turmoil was affecting their daily interaction.

No doubt these findings are only preliminary and that further studies are paramount to continue to explore not only why, but also when and how women become the victims of extreme violence. However, their message seems to be insightful, which is that a higher risk of being victimized and killed lies more in intense, though problematic, relationships, rather than in criminality itself.

## Discussion

This study focused its attention on women victims of deadly violence between 1970 and 2016 in Turin. Findings show that over these 46 years of investigation there has been a diminished rate of femicides. Nevertheless, they seem to suggest that most victims were not an indiscriminate and accidental target of violence.

These findings are counter-intuitive. While the common perception indicates that women are thought of as being at greater risk when away from home, with strangers, at night, and in isolated places, in fact most violence seems to happen at home or in familiar surroundings. Research shows that violence does not occur randomly, as shown in the description of the ≪ideal victim≫ and the ≪ideal offender≫ (Christie, 1986): victimogenic factors should be taken into account because they seem to be an essential component in fostering violence, transforming and escalating it into femicide. Understanding these aspects becomes relevant for preventive and intervention purposes.

According with international studies (Bailey et al., 1997), these data show that the victims involved in this study knew their perpetrators, and that the motives behind the killing mostly lie in problematic relationships and in family problems that led to aggressiveness, persecutory thinking, and then killing, and overkilling. They also show that IP femicide was more likely the result of a deteriorating relationship and of fading respect and trust between partners, than an act of pure cold crime; IPV was indeed the recurrent factor for escalating into femicide.

These data seem not to sustain the hypothesis of a gender violence to explain these cases of femicide, because in most of cases the killing was motivated by interpersonal and intimate motives that were at the basis of the escalation into deadly violence.

Current findings should be interpreted in the light of a few limitations that should be taken into account.

It was difficult to identify those cases in which the motive was purely gender - when the woman was killed because she was a woman. This motive might have been behind those cases in which victims and perpetrators did not know each other, but it was impossible, with these data, to reach that conclusion. In those cases in which the perpetrator was a total stranger, it could be assumed that the deadly violence had been motivated by control, power and antisocial motives. This is, in fact, related to the fact that some predatory murders were acted with coldness, detachment, and with some systematic precision, as in those cases in which the perpetrator committed more than a murder.

Because it was not possible to gather first-hand information from family members about the quality of the relationship between victim and perpetrator, and from perpetrators about the motives behind the killing the evidence gathered explain only part of dynamics of the IPV that fostered the femicide.

Furthermore, it was impossible, with these data, to reconstruct exactly whether the violence was mostly unilateral (from man toward woman), and to identify the recurrent victimogenic factors that interacted with other factors to escalate into femicide. In most cases in which this information was present it emerged that violence was a *by-product* of the relationship, was dyadic, and was the end result of a dysfunctional interaction in which personal, familial, cultural, economic and social aspects contributed to the worsening of the relationship. More studies are needed to explore the interactive combinations of criminogenic and victimogenic factors.

Despite these limitations, studies such as the one presented here are insightful in so far as they acknowledge that there is not a more suitable scientific subject such as IP femicide to see the gap between the ≪law on the books≫ and the ≪law on the streets≫ (Zimring and Hawkins, 1997).

Many years of under prosecuted IPV incidents may have fostered an implicit license that it is somehow tolerable to be abusive, insulting, psychological humiliating or physically coercive toward one's one partner, and this is why in such cases the problem cannot be “simply” identified with the “crime” committed, and solved by the administration of the law and the conviction of the perpetrator. This study shows that only a minority of the perpetrators were involved in a criminal career, and killed the victims because of pure criminality. What especially constituted the problem was the process of deterioration of the relationship, and the building up of a pattern of interpersonal violence that filled up with intolerance, misunderstanding, control and disrespect, the gaps that divided the partners.

Contrary to the media that presents a quite alarming situation (e.g., the *availability heuristic*) (Kahneman et al., 1982), these data show instead a reduction in the number of femicide over time, which is coherent with other psycho-criminological studies (Puzone et al., 2000; Dutton, 2012), and criminal data in Italy and in the Western world.

However, these findings should not be interpreted as if VAW and IPV were not a problem anymore or did not exist. Rather these findings inform us that the nature of abuse and violence has changed. Over the years, VAW and IPV may have taken a more silent (e.g., psychological and emotional) and a less spectacular (e.g., killing) vest. This study also raises some further questions that deserve a specific investigation over whether changing life or breaking off contacts with the perpetrator does really interrupt victimization or whether it instead increases the risk of it. It may be that previous forms of violence and victimization, especially within dysfunctional relationships, remain a determining factor of continual abuse, though differently manifested. Certainly, many women continued being abused, also after distancing themselves from their perpetrators; this is so because abusive and violent relationships last a long time, even though they change shape, and proximity.

## Conclusion

Despite all the emancipation characterizing Western society, women are still the target of IPV. In so many cases this violence is less physical, with the result that the process of escalation might be transformed, so that women are less likely to be killed, or if killing emerges, it occurs after a longer time of silent, “behind curtains”, abuse in which they had been dominated and controlled.

What is the message to take home from this study? VAW and IP femicide require an interdisciplinary perspective. Scientific research advocates a multidimensional approach, which looks at the problem with an *equifinality lens*. Femicide cannot be seen only as the story of cultural violence against women, because the story of the deadly violence against the women involved in this study, as in many other studies around the world, recounts more a story of IPV; the unfortunate story of a specific victim in a specific relationship who became the attention for violent vent out and destructiveness. This differentiation is essential to avoid reducing or enlarging to culture something, which is instead, according to these data, especially relational, at times intimate, at times familial, and at other times social, though essentially all of these levels together.

In addressing this differentiation in this research, the interest was to avoid the Hamletian's error, which is imagining the story of Hamlet without Ophelia, though Ophelia could easily disappear because her story was thought as ancillary or as irrelevant without Hamlet (Edwards, 1979).

These findings demonstrate that most victims (Ophelias) and perpetrators (Hamlets) exist the way they are because of the way they interact, or do not interact, or interact with each other dysfunctionally, disrespectfully, and immaturely.

## Ethics statement

This study was carried out in accordance with the recommendations of the Italian Code of Psychologists and the APA Code of Ethics. The protocol was approved by the Bioethic Committee of the University of Turin. All data gathered and employed in this study were anonymized and made it unidentifiable according to the Italian privacy and data protection law that regulates the use of data for scientific research.

## Author contributions

GZ and SG conceived, planned, organized, designed the study, and attained data. GZ analyzed the data and interpreted the results. GZ drafted the article. GZ and SG critically revised of the article.

### Conflict of interest statement

The authors declare that the research was conducted in the absence of any commercial or financial relationships that could be construed as a potential conflict of interest.

## Footnotes

^1^Calculating the costs of VAW is complicated because studies employ different methods of estimation, gather different data on different types of violence (intimate partner or domestic violence), count various types of costs (to direct and indirect victims, employers, the community, and governments) (Council of Europe, 2012). A report by Badalassi et al. (2013) entitled “Quanto costa il silenzio?” (that means “What is the cost of silence?”), published by the Italian NGO Intervista Onlus (and from now on quoted as Intervista Onlus), attempts to offer accurate estimates of the costs. The study concludes that the overall indirect (non-monetary) and social multiplier costs of violence against women in Italy is about EUR 14.3 billion (approximately USD 16.2 billion) (Intervista Onlus, 2013, p. 15). According to the same study, when the direct costs to the public sector (EUR 1.8 billion) (USD 2.1 billion) and the economic multiplier costs (EUR 604 million) (USD 690 million) were added, the total was estimated at EUR 16.7 billion (USD 19.19 billion). The study also shows that the costs related to the prevention and contrast of violence against women would be around EUR 6.3 million (USD 7.2 million) (Intervsita Onlus, 2013, p. 15).

^2^Taking into account all the costs briefly described, different laws were passed in the world to regulate VAW. In 2011, the Europe Council ratified the Istanbul Convention (2012) that was the first comprehensive tool for opposing all forms of violence. In Italy, a legal enforcement process against discrimination was achieved, passing through different steps (as New Family Law, Divorce Law and Abolition of Crimes of Honor) and in 2008, with the enactment of the law known as “Measures against persecutor acts,” some significant measures were set up as the preliminary sign of significant social and legal changes. In 2013, Italy ratified the Istanbul Convention (2012). Moreover, the Italian government passed an array of measures designed to tackle the problem of violence against women, and introduced Law Decree n. 93 of 14 August 2013, which then converted, with amendments, into Act n. 119 of 15 October 2013. This law was enacted by laying down some key issues: complaint irrevocability; legal aid to victims; increased punishment in special cases (e.g., victim's pregnancy, presence of children, etc.); the possibility for the alleged victims of reporting anonymously to the police their claims over IPV incidents; the introduction of a warning procedure in the case of stalking. Legal initiatives were complemented by the activity of anti-violence centers and women's associations, springing up on Italian territory in the last 20 years, with the aim to fill gaps in the welfare body.

^3^Many risk assessment tools, in the fashion of professional structured instruments are available in the scientific community for professionals (see Zara and Farrington, 2016 for a complete review). For instance, a more general assessment of threat can be performed with the *Danger Assessment* (DA) (Campbell, 1995). For a more targeted assessment the *Spousal Assault Risk Assessment Guide* (SARA) (Kropp et al., 1995, 1999) is the most used. For a more specific assessment of obsessive search for intimacy risk, *Stalking Assessment and Management* (SAM) (Kropp et al., 2008) is currently one of the best instrument available.
